# Pre-Clinical Assessment of Novel Multivalent MSP3 Malaria Vaccine Constructs

**DOI:** 10.1371/journal.pone.0028165

**Published:** 2011-12-01

**Authors:** Gilles Bang, Eric Prieur, Christian Roussilhon, Pierre Druilhe

**Affiliations:** Malaria Vaccine Development Laboratory, Institut Pasteur, and Vac4all initiative, Paris, France; Tulane University, United States of America

## Abstract

**Background:**

MSP3 has been shown to induce protection against malaria in African children. The characterization of a family of *Plasmodium falciparum* merozoite surface protein 3 (MSP3) antigens sharing a similar structural organization, simultaneously expressed on the merozoite surface and targeted by a cross-reactive network of protective antibodies, is intriguing and offers new perspectives for the development of subunit vaccines against malaria.

**Methods:**

Eight recombinant polyproteins containing carefully selected regions of this family covalently linked in different combinations were all efficiently produced in *Escherichia coli*. The polyproteins consisted of one monovalent, one bivalent, one trivalent, two tetravalents, one hexavalent construct, and two tetravalents incorporating coiled-coil repeats regions from LSA3 and p27 vaccine candidates.

**Results:**

All eight polyproteins induced a strong and homogeneous antibody response in mice of three distinct genotypes, with a dominance of cytophilic IgG subclasses, lasting up to six months after the last immunization. Vaccine-induced antibodies exerted a strong monocyte-mediated *in vitro* inhibition of *P. falciparum* growth. Naturally acquired antibodies from individuals living in an endemic area of Senegal recognized the polyproteins with a reactivity mainly constituted of cytophilic IgG subclasses.

**Conclusions:**

Combination of genetically conserved and antigenically related MSP3 proteins provides promising subunit vaccine constructs, with improved features as compared to the first generation construct employed in clinical trials (MSP3-LSP). These multivalent MSP3 vaccine constructs expand the epitope display of MSP3 family proteins, and lead to the efficient induction of a wider range of antibody subclasses, even in genetically different mice. These findings are promising for future immunization of genetically diverse human populations.

## Introduction

Malaria caused by *Plasmodium falciparum* (*P. falciparum*) is one of infectious diseases with the greatest impact on worldwide public health with morbidity figures of 300–500 million and mortality reaching nearly one million per year. Control of the disease has become difficult with increasing drug-resistant forms of parasites, resistance of *Anopheles* mosquito vectors to insecticides and global warming. A safe and affordable malaria vaccine would offer the most cost-effective tool to curb this situation.

Several vaccines aimed at rolling back malaria are currently at various stages of development. Among blood stages proteins considered as vaccine candidates, antigens expressed by merozoites have emerged as the most promising vaccine candidates. The merozoite surface protein 3 (MSP3.1) was selected based on immuno-clinical analysis of natural *P. falciparum* and human host interactions [1]. The value of MSP3.1 as a vaccine candidate was reinforced when it was found that the C-terminus domain was highly conserved among various *P. falciparum* field isolates from Africa and Asia [2], [3]. A 69 amino acids (aa) region in the C-terminus region displayed promising features for the development of a subunit vaccine in several studies involving malaria exposed individuals and malaria naïve adults enrolled in a phase I trial [4], [5], [6], [7], [8], [9]. Results showed that a MSP3-long synthetic peptide (MSP3-LSP) vaccine formulation combining conserved epitopes from MSP3.1-CT elicited high humoral and cellular immune responses in human volunteers. The B-cell response was primarily constituted of cytophilic IgG subclasses (IgG1 & IgG3) which were effective at achieving parasite killing *in vitro* in cooperation with blood monocytes, and which were also found associated with protection against malaria attacks in individuals from endemic areas [10]. Recent results moreover show that this construct can induce protection against clinical malaria in young African children [9].

We have observed that *msp3* belongs to a multigene family with unusual features, which distinguish it from all other *P. falciparum* multigene families such as *var* and *rifin*. The new multigene family located on chromosome 10, encodes for six merozoite surface proteins that share the same structural organization of their C-terminal regions. They are simultaneously transcribed and expressed on the merozoite surface. They include regions with sequence homologies flanked by regions that markedly differ from one family member to the other. Their full sequence conservation among distinct isolates is striking and most unusual [11]. Antigenicity analysis of this MSP3 multigene family using 6 C-term recombinant proteins and 36 peptides derived from them (i.e. 6 epitopes covering regions “a” to “f” from each C-T) showed that all peptides were antigenic, with a distinct IgG isotype pattern for each, though with an overall predominance of the IgG3 subclass. Human antibodies affinity-purified upon each of the 36 peptides exerted an anti parasitic activity through an antibody-dependent cell inhibition (ADCI) in cooperation with blood monocytes. Region covering epitopes “b” to “d” displays the highest homology among the six proteins and generates a broad network of antibodies cross-reactive with the remaining proteins with various avidities [12]. In addition, within non-homologous regions, sequence divergences from one gene to the other, were thought to be related to T-helper function, and this was documented in MSP3.1, each sequence being potentially better fitted to a given MHC class-II subset. The genetic conservation of this diversity would possibly provide improved T-cell help in individuals with diverse MHC class-II genetic background [13]. Conversely, immunogenicity studies indicated that certain regions are involved in the regulation of the intensity of immune responses: *i.e.* removing the “e–f” region from MSP3.1 resulted in an increase by two orders of magnitude of antibodies to the ADCI-relevant “b–d” region [13]. We have argued previously that this conservation of homologous and divergent regions could contribute to generate a wider range of diversity in affinity, avidity and fine-specificity in the antibody repertoire [11]. This might result in reactivity to a wide range of original and related epitopes and lead to greater efficacy of *in vitro* growth inhibition of parasite in the ADCI.

At the origin of the present work is the hypothesis that, by increasing the number of protective epitopes delivered in a vaccine formulation, more balanced and better-targeted responses would be generated in a larger range of immuno-genetically diverse population. Therefore, we designed eight new and different chimerical vaccine constructions, by combining homologous and non-homologous sequences derived from each of the six *msp3* genes. The design of each vaccine construct was based on information gathered previously about: i. Structural organisation, sequence and conservation of each family member [11]; ii. Detailed antigenicity data gathered in endemic area populations using 36 peptides derived from the 6 proteins [12] and iii. Immunogenicity data obtained in mice [13].

In the present study we evaluated the immunological properties of these eight polyproteins with regard to three main criteria: **a)** Immunogenicity in 3 mouse strains; **b)** Antigenicity in sera from endemic area individuals (Ndiop, Senegal); **c)** Ability of vaccine-induced antibodies to recognize native parasite antigens and exert *in vitro* an anti parasitic activity in ADCI. This preclinical approach was aimed at selecting the constructions recognized by functional antibodies naturally produced by malaria-exposed human beings and inducing broad, diverse antibody responses, consisting of antibodies efficient in MN-dependant parasite killing.

## Materials and Methods

### Ethics Statement

Procedures and experiments involving mice were approved by Institut Pasteur Safety Committee and performed in accordance with French legislation in general and in particular with Institut Pasteur Ethical Committee guidelines for animal handling warranted by the Approval Number A7515-27. Human blood samples from healthy malaria naive volunteers were sampled at the Etablissement Français du Sang (EFS, Paris) and used in accordance with French legislation in general and in particular with a convention between Institut Pasteur and EFS as licensed by Approval ID HS2003-3251.

### Parasites

Asexual blood stage parasites of *Plasmodium falciparum* (3D7 clone) were cultured in complete medium (CM) composed of RPMI 1640 with L-Glutamine (Invitrogen) supplemented with 5 mg Hypoxanthine, 35 mM HEPES, 23 mM NaHCO_3_ and 0.5% Albumax I (Invitrogen) in the presence of AB^+^ erythrocytes. Cultures were maintained at 37°C in a humidified 5% CO_2_ incubator. When required, ring stage or schizont stage parasites were enriched respectively either by 5% sorbitol treatment (Acros Organics) or by flotation on 1% porcine skin gelatin type A (Sigma-Aldrich).

### Antigens

The recombinant antigens used in this study corresponded to the C-terminal part of the *P. falciparum* (strain 3D7) MSP3 proteins (MSP3-CT); (MSP3.1_167–354_; PlasmoDB ID: PF10_0345, MSP3.2_161–371_; PlasmoDB ID: PF10_0346, MSP3.3_228–424_; PlasmoDB ID: PF10_0347, MSP3.4_508–697_; PlasmoDB ID: PF10_0348, MSP3.7_214–405_; PlasmoDB ID: PF10_0352, MSP3.8_537–762_; PlasmoDB ID: PF10_0355). The recombinant antigens corresponding to LSA1 (*P. falciparum* T9/96 clone, GenBank ID: Z30_320.1) and LSA3-GST729 (*P. falciparum* T9/96 clone, GenBank ID: AJ007011) were produced as previously described [14], [15]. The p27 peptide (PlasmoDB ID: PFF0165c) corresponding to amino acids AA_845–871_ from a protein targeted by the antibody-dependent parasite growth inhibition mechanism (ADCI) was a gift from Pr. Giampietro Corradin (University of Lausanne, Switzerland) [16].

### MSP3-derived polyprotein (PP) immunogens

**Figure 1:**
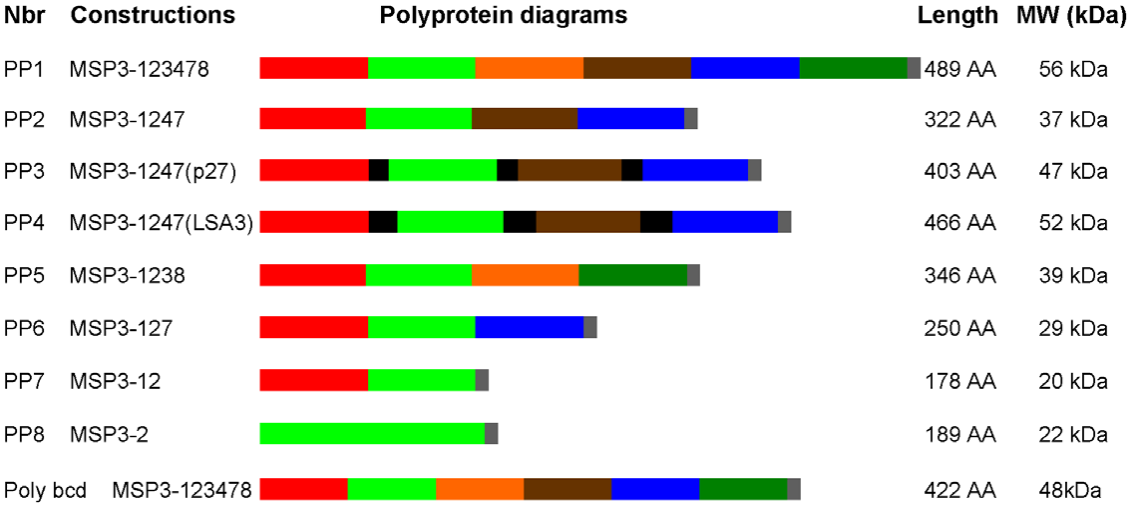
Diagrammatic representation of the MSP3-based PPs. The antigens in the PP constructs are colour-coded as follow; MSP3-1 (AA 184 to 251) in red, MSP3-2 (AA 161 to 257) in light green, MSP3-3 (AA 228 to 307) in orange, MSP3-4 (AA 508 to 579) in dark brown, MSP3-7 (AA 214 to 285) in dark blue, MSP3-8 (AA 537 to 624) in dark green. The construction PP8 is constituted by the assembly of the amino acid sequences 161–257 to 293–371 of MSP3-2. The amino acid sequences in the PPs correspond to the sequences of the clone 3D7 of *P. falciparum*. PP3 and PP4 contain p27 and LSA3 linkers respectively and are quoted in black on the diagram. The histidine tag appears in gray at the C-terminal extremity. The poly-bcd polyprotein construct designed previously [13] which incorporates all the “b–d” regions of the six MSP3-CT antigens is also shown, for comparison. The size and theoretical molecular weight of the polyproteins produced in *E. coli* are indicated.

In the following, MSP3-1 to MSP3-6 refers to the 6 proteins belonging to the MSP3 multigene family, whereas the polyproteins PP-1 to PP-8 refer to the constructs assembling different fragments from the MSP3 proteins (Figure 1). They are called “PP” to make it easier for the reading. Nucleic acid sequences coding for the MSP3 polyproteins (PPs) were chemically synthesized (GenScript). The MSP3 amino acid sequences covalently linked in the 8 PP constructs (Figure 1) were selected for their antigenic and immunological properties as well as for their absence of polymorphism. They comprised MSP3.1 AA_184–251_, MSP3.2 AA_161–257_, MSP3.3 AA_228–307_, MSP3.4 AA_508–579_, MSP3.7 AA_214–285_, and MSP3.8 AA_537–624_. PP1 contains all the members of the MSP3 family. PP2 containing MSP3-1, -2, -4 and -7 was chosen to assess the effect of sequences forming coiled-coil alpha helices inserted between the MSP3 domains on its production and immunogenicity. Therefore, peptide p27 described above was inserted in PP3, whereas PP4 contained a combination of the most common tetrapeptide repeated units in the LSA3 AA_223–586_ region [15] (PlasmoDB ID: PFB0915w). PP5 contains MSP3-1, -2, -3 and -8, PP6 contains MSP3-1, -2 and -3 and PP7, MSP3-1 and -2. The last construction (PP8) corresponds to a shortened version of MSP3.2-CT where an embedded glutamic acid-rich region was deleted by linking the sequences coding for AA_161–257_ to AA_293–371_.

### Production of the recombinant proteins in *Escherichia coli*

The immunogens used for the vaccination and antigens used in the *in vitro* tests were produced in the BL21 strain of *E. coli* (Invitrogen) with a C-terminal His-tag. The bacteria were grown to a steady state phase and diluted 20-fold in fresh culture media. The production of the recombinant proteins was induced with IPTG (10 µM) when the bacteria culture reached an optical density at 600 nm of 0.6. The bacteria were recovered after two hours and a half of production in 25 ml of a lysis buffer (LB) composed of 6M urea, 300 mM NaCl, 46.6 mM Na_2_HPO_4_, 3.4 mM NaH_2_PO_4_, and 10 mM imidazole and then lysed on ice by ten one minute-long sonication pulses spaced by one minute resting time. The lysate was cleared of debris by centrifugation (10 000 g, 10 min, 4°C) and the supernatant passed through a 0.45 µm filter unit (Millipore). The recombinant proteins were purified by affinity on 5 ml of a nickel-charged resin (Qiagen). After washing the column with 10 ml of LB followed by two washes with LB containing 20 mM of imidazole, the recombinant proteins were eluted in 10 ml of LB with 250 mM of imidazole. The eluted proteins were placed in membrane tubing with an 8 kDa of molecular weight cut-off (Spectrum Laboratories) and dialyzed against 5 liters of phosphate buffered saline (PBS) overnight at 4°C. The protein solutions were concentrated by ultra filtration on membranes with a 10 kDa cut-off (Sartorius) and passed through 1 ml of endotoxin removal resin with immobilized polymixin B (Pierce). The endotoxin level in the protein preparations was assessed with the PyroGene® kit (Lonza) according to the manufacturer instructions. Solutions negative for the assay (sensitivity of 0,01 EU of endotoxin per ml) were considered free of endotoxins. The protein concentration was measured by the Bradford method (Bio-Rad) against a dilution range of bovine serum albumin and adjusted to 1 mg/ml with endotoxin-free PBS. They were stored at −20°C until use.

### Mice and immunization protocol

Two inbred strains, C57BL/6 (H-2b) and BALB/c (H-2d) and one outbred strain (Swiss), of four-week-old female mice were obtained from Elevage Janvier (Le Genest-Saint-Isle, France). For each mouse strain, the animals grouped by PP immunogen (n = 7) received every two weeks three immunizations and sera were collected after the second and the third. In view of the deleterious effect of repeated immunizations observed previously using the MSP3.1 C-terminus construct, which was not observed using the MSP3-LSP, all mice received thereafter three additional immunizations to analyze effects of cumulative immunizations [13]. All successive immunizations were performed by subcutaneous injection of 20 µg of recombinant PP emulsified in 70% Montanide ISA720 (Seppic, France) in a volume of 100 µl. Control groups received adjuvant alone or the pre-erythrocytic stage antigen, LSA1. Immune sera were collected from tail blood two weeks post injection from the second and up to the sixth injection and stored at −20°C until tested.

### Western blotting

Proteins from 6×10^7^ schizont-infected erythrocytes were separated by electrophoresis in a denaturing 10% polyacrylamide gel and electro transferred on a nitrocellulose membrane (Amersham Biosciences). After saturation in PBS-5% skimmed milk (diluent buffer), membrane strips were cut and incubated for one hour at room temperature on a rocking tray with pool of immune sera from each immunization group tested at a 1∶1000 in diluent buffer. Strips were incubated for one hour with the secondary antibody, a goat anti-mouse IgG conjugated with alkaline phosphatase at 1∶7500 in diluent buffer, and then incubated in BCIP/NBT color substrate solution (both from Promega). Uninfected erythrocytes and sera from adjuvant alone immunized mice were used as negative controls.

### Malaria-exposed individuals from Ndiop village

Antigenicity of each PP construct was assessed with sera sampled from individuals living in Ndiop village (Senegal, West Africa), a malaria mesoendemic area, described elsewhere [17]. Sera from individuals living in Ndiop, where obtained after using stringent protocols of clinical follow-up consisting of daily surveillance by a medical staff (present 24 hours a day, seven days a week) in order to identify and to analyse each single episode of morbidity [18], [19]. The villagers received between 10 and 60 infected bites per person per year, and in children between 1 and 11 years of age, 2 or 3 episodes of clinical malaria were detected each year. A thick blood smear was Giemsa-stained and examined immediately for the purpose of treatment when a report or complaint of fever, headache or vomiting, were observed by a trained nurse residing permanently in the village. Irrespective of age, a parasite density threshold corresponding to a trophozoite to leukocyte ratio above 0.5, i.e. roughly, a circulating blood parasitaemia level of slightly less than 0.1%, or 5000 parasites/µl of blood, associated with clinical signs of sickness was considered as a malaria attack. This diagnostic was confirmed when the episode of illness disappeared after appropriate anti-malaria treatment. The percentage of time really spent in the village by the inhabitants tested in the present study was 88.5% during the first year and 78.3% during the entire clinical follow-up. Malaria attacks were detected in 64.3% of the individuals who experienced a mean number of 2.61±1.79 episodes during the first year of follow-up and 5.39±3.74 clinical attacks during the entire survey.

The informed consent of each villager (or that of the parents in the case of children) was obtained at the beginning of the study and was renewed at the beginning of each year of the survey. The study design received clearance from the national Senegalese ethical committee.

### Enzyme Linked Immunosorbent Assay (ELISA)

Antibody responses against the six MSP3-CT peptides (i.e. MSP3.1; MSP3.2; MSP3.3; MSP3.4; MSP3.7; MSP3.8) were determined after each immunization with the PP constructs in ELISA assays as described elsewhere [20]. 96-well polystyrene plates (Costar) were coated overnight at 4°C with each MSP3-CT peptide at 2.5 µg/ml in PBS. After saturation, plates were incubated with mice immune sera in PBSM-0.05% Tween 20 (diluent buffer) at a 1∶1000 dilution. This dilution was used because it allowed comparison of antibody responses of the present generation of MSP3-derived constructs with the previous one [13]. IgG and IgM antibody responses were revealed with a horseradish peroxidase-conjugated goat-anti-mouse secondary antibody (GAM-HRP) (Caltag Labs, Invitrogen) in diluent buffer at 1∶3000 and 1∶2000 dilutions, respectively. In addition, murine IgG subclasses were measured with isotype-specific GAM-HRP secondary antibodies diluted 1∶4000 (IgG1, IgG2a, IgG2b), 1∶2000 (IgG3) (Caltag Labs, Invitrogen) and 1∶4000 (IgG2c) (Beckman Coulter) in diluent buffer. The colorimetric reaction was triggered by the adjunction of a TMB substrate solution (Uptima, Interchim) and stopped with 1 M H_3_PO_4_ before reading of the optical density at 450 nm (OD_450_). Determination of IgG titers in murine sera was done from ten fold serial dilution (starting to 1∶1000). A mean OD_450_ value = 0.2 was the end point titer considered for IgG titer determination. For other murine data, the results were expressed as the geometric mean of the OD_450_ obtained with single dilution at 1∶1000 of murine sera. The murine and human cytophilicity ratios (CR) were calculated according to the following formulas; ((IgG2a+IgG2b+IgG2c)/(IgG1+IgG3+IgM)) and ((IgG1+IgG3)/(IgG2+IgG4+IgM)), respectively. The CR value was considered positive if >1, meaning thereby an isotypic imbalance in favour of cytophilic IgG subclasses.

Briefly, the total IgG, IgM, and the IgG subclasses levels were assessed in 1∶100 diluted sera as described elsewhere [20]. Results were expressed as OD_450_ ratios, which were calculated by dividing the mean OD_450_ of test sera by the mean OD_450_ value+three standard deviations of the mean obtained from healthy donors. A serum was considered positive if this ratio was higher than 1.

### Immunofluorescence assay (IFA)

The ability of mice immune sera to recognize native parasite proteins on air-dried and acetone fixed thin smears of the blood stage of *P. falciparum* (3D7 clone) was assessed by IFA as previously described [21]. IFA and ADCI assays were performed the same day, in order to check the antibody content of the sera after frozen storage, and immediately before the functional assay.

### Preparation of human blood monocytes

Peripheral blood mononuclear cells (PBMC) from healthy blood donors were separated by Ficoll-Hypaque (P.A.A. GmBH, Germany) density gradient centrifugation, washed in Ca^2+^ and Mg^2+^ free HBSS buffered with 10 mM HEPES (Invitrogen). Cells were frozen in heat-inactivated AB^+^ human serum with 10% DMSO (Sigma-Aldrich) at a final concentration of 15×10^6^ cells/ml and stored in liquid nitrogen until use. The phenotype of the monocyte (MN) sub-population was monitored by flow cytometry. Briefly, 10^6^ cells were incubated in 100 µl of PBS-5% FCS (FACS buffer) in the presence of 0.1 mg/ml of anti-CD14-PE and anti-CD16-PC5 (Beckman Coulter) for 30 min at 4°C in the dark. At least 5.10^4^ cells were acquired and then analyzed by flow cytometry (FACSCalibur, BD Biosciences).

### Antibody-dependent cellular inhibition assay (ADCI)

The ADCI assay previously described elsewhere [22], [23] was carried out with serial dilutions (2.5% - 0.5% - 0.1%) of pooled sera from each PP-immunized mice groups corresponding respectively to 1∶200, 1∶40, and 1∶8 dilutions of the IFA final antibody titre. A pool of sera from 180 hyperimmune African adults (PIAG) was employed at 10% final (which corresponds to a concentration of 2 mg/ml) as a positive control to assess the reproducibility between each assay. A number of PBMC containing 2.10^5^ MN, according to flow cytometry phenotype analysis, was added per well of a 96-wells flat bottom sterile plate. MN were further selected by adherence after incubation for one hour at 37°C, in a 5% CO_2_ atmosphere. An asexual blood stage parasite culture with very mature schizonts (0.5% parasitemia, 2.5% haematocrit) was added on the MN monolayer previously prepared. Murine test and control pooled sera were added according to the serial dilutions described above. The intrinsic anti-parasitic effect of the sera (control and test sera) was assessed in wells containing the blood stages parasites without MN. Plates were incubated in a candle jar at 37°C, in a 5% CO_2_ incubator. At 48 and 72 hours, 50 µl of CM was added to each well. At 96 hours the assay was stopped and the parasitemia determined by flow cytometry (FACSCalibur, BD Biosciences).

### Flow cytometry

The enumeration of erythrocytes infected by viable malaria parasites was done by flow cytometry through a double staining method using hydroethidine (HE) and thiazole orange (TO) (Sigma-Aldrich) [24]. Briefly erythrocytes were incubated for 20 min at 37°C in the dark with 20 µg/ml of HE diluted in PBS-1% FCS (FACS buffer), washed three times in FACS buffer, followed by a 30 min incubation at room temperature in the dark with TO diluted at 1∶15000 in FACS buffer. Analyses were performed on 1.10^5^ erythrocytes with the CellQuest Pro® software. The parasitemia was determined as the percentage of double stained infected erythrocytes among the whole erythrocyte population. The specific growth inhibitory index (SGI) was calculated according to the following formula: SGI = 100×[1−(percent parasitemia with MN and mice test sera/percent parasitemia with mice test sera)/(percent parasitemia with MN and naïve mice sera/percent parasitemia with naïve mice sera)]. Based on results previously obtained with a range of negative and positive controls (unpublished results), the threshold of positivity in ADCI is >30% SGI. To take into account experimental variations results are reported as Adjusted-SGI relative to the positive reference control employed in each ADCI plate.

### Statistical analysis

Results were tested in univariate analyses performed with the Mann-Whitney *U* test. Available information pertaining to the IgG isotype responses specific for the different polyproteins was also analyzed in multivariate models. The potential association between anti-MSP3 cytophilic responses (i.e. cytophilicity ratios) and the occurrence of malaria attacks was tested with the JMP® software (SAS Institute). We applied a stepwise regression model in which the effect of age and that of all other confounding variables suspected of having a potential impact on the occurrence of malaria attacks was included (Hb phenotype, G6PD deficiency and the time actually spent in the village). The explanatory variables tested were selected because of our existing knowledge of the potential involvement of these different parameters (with reference to published papers and to our previous observations in different malaria endemic areas). The relationship between the variable to explain (the number of clinical episodes of malaria) and the available explanatory variables was tested and the variables that were identified as non-significant were eliminated. The criterion for elimination was that a predictor variable was excluded when its partial regression coefficient failed to be significant at the 0.1 level. The *F* ratio was the test statistic used to determine whether the model as a whole had statistically significant predictive capability in the regression framework. Departures from the null hypothesis tended to give values of *F* ratios that were greater than unity. The null hypothesis was rejected when the *F* ratio was large and the associated *p* values were low. Since we used at least eight successive tests (one per construct) to look for the possible association between antibody responses and the number of malaria attacks identified over one and three years periods after the blood sampling, we decided to consider a value of *p*<0.0031 as significant (Bonferroni correction).

## Results

### Production of PPs in *E. coli*

The eight chimerical PPs were readily produced in *E. coli* with similar yields ranging from three to five milligrams per litre of culture. The recombinant products that were purified by affinity chromatography showed the expected experimental molecular weights for polypeptides ranging from 178 to 489 amino acids without detectable degradation products (Figure S1A). The immunological specificity of the recombinant PPs was confirmed by western blotting with an anti-MSP3.1 rat immune serum (Figure S1B).

### PPs are immunogenic and induce cross-reactive antibodies against the proteins from the MSP3 family

**Figure 2:**
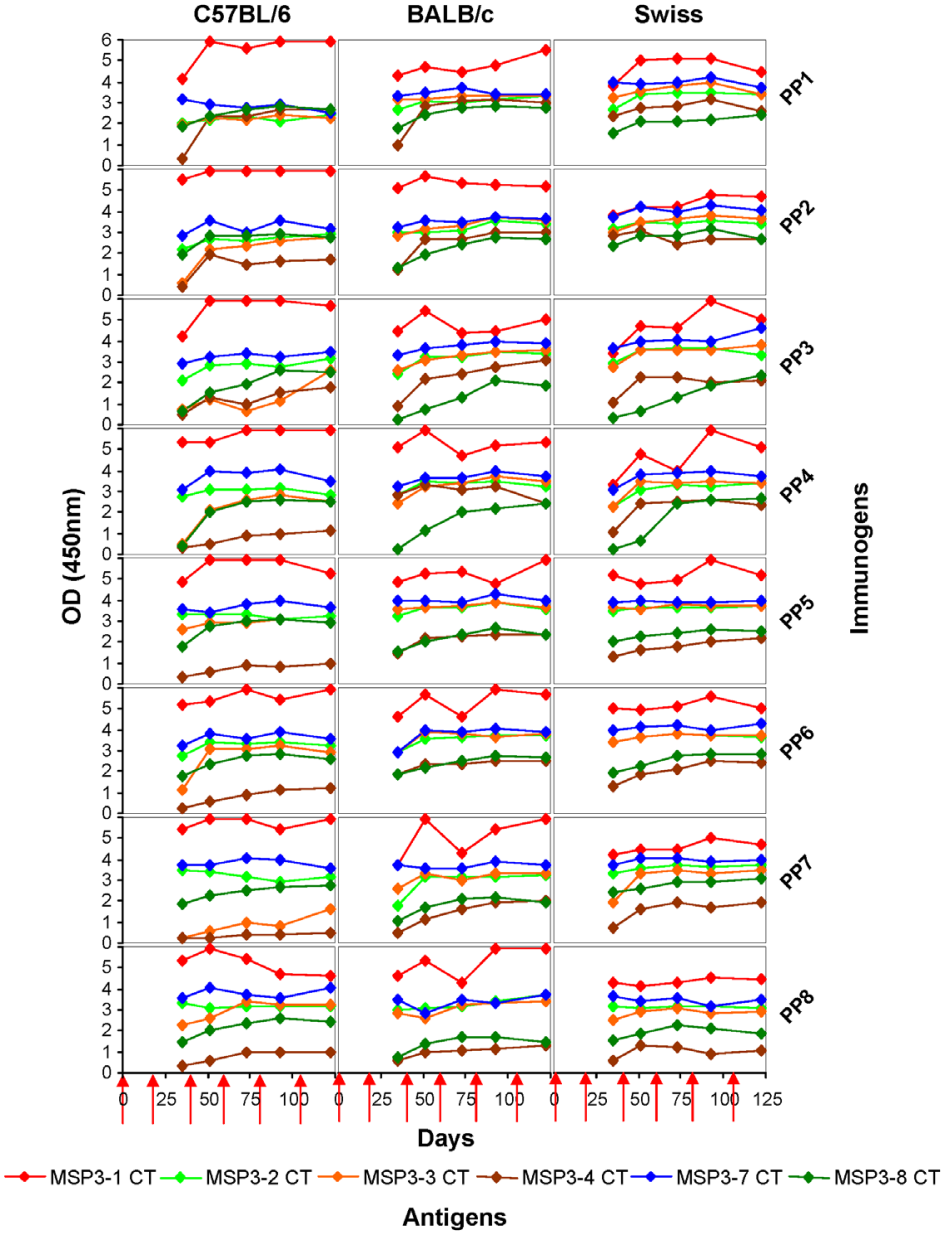
Time course of specific PP-induced IgG against MSP3-CT antigens. Kinetic of immunogenicity of PPs against each of the MSP3-CT antigen was investigated in mice of three distinct genotypes. Total IgG response was measured every two weeks after each immunization and expressed as mean OD_450_ value. The arrows on the x-axis indicate the timing of the subcutaneous injections of 20 µg of recombinant PPs in Montanide ISA720 adjuvant. In the particular case of MSP3-1 values are only indicative as they are higher than OD = 5, i.e. than the linear range of the reader.

The magnitude and kinetics of the antibody responses induced by the PP constructs mounted against each MSP3-CT antigen were assessed in three mouse strains (C57BL/6, BALB/c and Swiss). Results show that the IgG responses against most of the antigens reach a maximum after two immunizations independent of both the immunogen employed, and the genetic background of mice, suggesting a minimal implication of the host genetic background on the induction of the antibody response against the MSP3-CT proteins (Figure 2). Responses to other antigens from the MSP3-CT family, not included in the immunogens, developed later, such as for example against MSP3.8-CT in animals immunized with the MSP3.1247-based constructions (PP2, PP3 and PP4), where the response reached a plateau after three to five injections. These delayed responses may reflect a qualitative change over time - a maturation of immune responses - which progressively extends to cross-reactive epitopes not included in the PPs.

**Figure 3:**
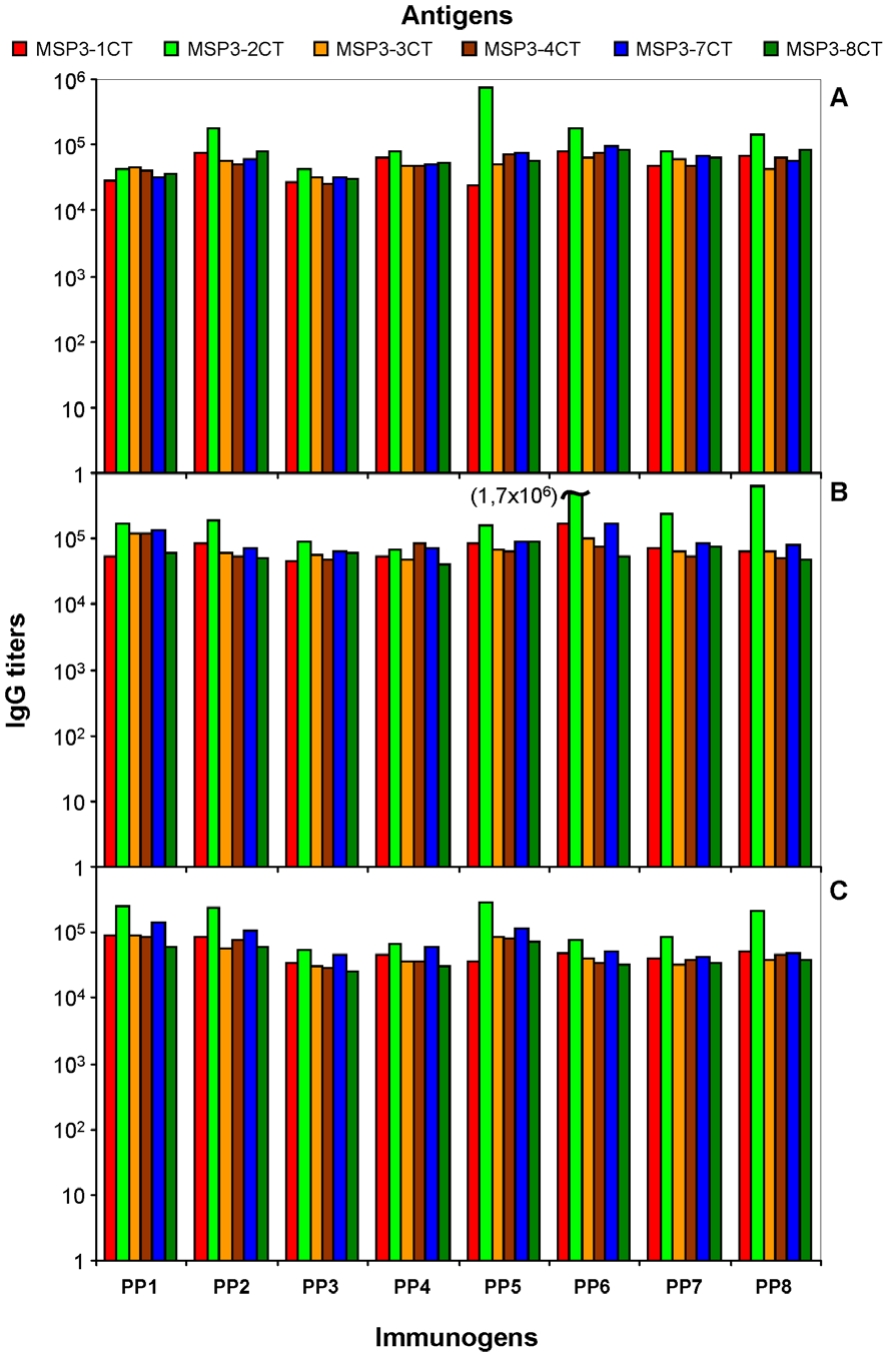
Immunogenicity of the PPs against MSP3-CT antigens. Total IgG titers were measured two weeks after the third immunization of C5BL/6 (A), BALB/c (B) and Swiss (C) with MSP3 polyprotein constructs. IgG titers for LSA1 or adjuvant immunized mice, not represented here, against MSP3 family antigens were insignificant. End point for IgG titer determination was a mean OD_450_ = 0.2.

The addition of repeated motifs from LSA3 and p27 forming coiled-coil alpha helices that are unrelated to MSP3 between the members of MSP3.1247 (PP3 and PP4) did not lead to any detectable change in the profile of the immune response, suggesting that they do not contribute to modify the immunogenicity of the MSP3 antigens as compared to PP2. Mice that received controls immunogens such as adjuvant alone or an irrelevant antigen (LSA1) did not develop any detectable specific anti-MSP3 response. Finally, over-immunization by delivering 3 boosting doses after the third immunization did not result in any significant decrease in antibody titers related to the induction of possible immune-regulatory mechanisms, as had been observed using MSP3.1-CT [13]. Antibody titers observed with the present PP constructs, against MSP3-CT antigens after the third immunization, were higher and more homogenous (Figure 3) than titers obtained when using the preceding generation of PP construct that was made by combining domain covering the “b–d” region from each MSP3-CT antigen extensively described elsewhere [11], [12], [13].

### Murine cytophilic IgG are predominant subclasses after immunization with PPs

**Figure 4:**
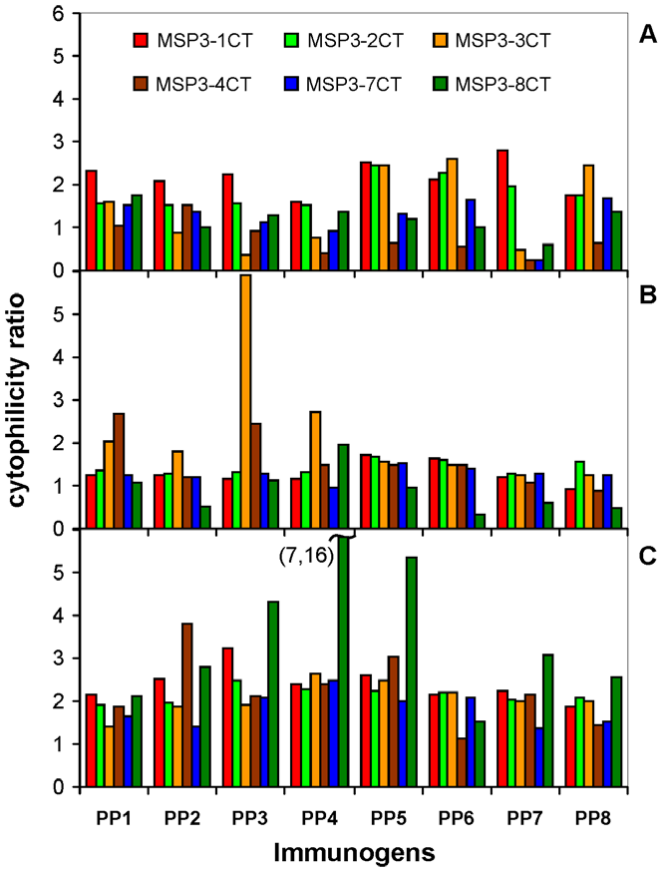
PPs induce a dominance of murine cytophilic IgG against MSP3-CT antigens. Cytophilicity ratio (CR) of IgG response against MSP3-CT antigens was assessed in C57BL/6 (A), BALB/c (B) and Swiss (C) mice, two weeks after the third immunization with PPs. CR was measured as following (IgG2a+IgG2b+IgG2c)/(IgG3+IgG1+IgM). Results were expressed as the geometric mean of the OD_450_ obtained for the single dilution of sera at 1∶1000.

A correlation between the progressive acquisition with age of cytophilic antibody isotypes directed against *P. falciparum* specific antigens (such as the members of the MSP3 family) and protection against clinical malaria had been identified in several immuno-epidemiological studies [10], [25], [26]. We evaluated the potential of the newly designed PPs to induce such isotype imbalanced responses in sera of immunized mice by measuring the cytophilicity ratio (CR). The cytophilic response against all MSP3-CT antigens was predominantly of the IgG2a isotype in BALB/c and Swiss strains (Figure S2), whereas the IgG2c isotype was predominant in the C57BL/6 strain (Figure S3). Indeed, it has been established that mice with a B6 genetic background preferentially express antibodies of the cytophilic IgG2c isotype instead of the IgG2a [27], [28], [29], [30]. Further support for the preceding statement is evident from the fact that, specific IgG2b response against each MSP3-CT antigen was similar in these three murine distinct genotypes. Among the non cytophilic responses produced, the IgG1 isotype prevailed over the IgG3 and IgM subclasses in the three strains of mice (data not shown). In agreement with these results, CR were comparable with few exceptions, whatever the immunogen and the MSP3-CT antigen analyzed. Values of CR were generally greater than one in the inbred C57BL/6 and BALB/c (respectively, Figure 4A and 4B) and in outbred Swiss (Figure 4C) mice. Some PPs resulted in particularly high CR (CR>3) as for example, antibodies induced by PP3 against MSP3.3-CT in BALB/c (Figure 4B), or antibodies induced by PP2 against MSP3.4-CT, or those induced by PP3 against MSP3.1-CT, or by PP3, PP4 and PP5 against MSP3.8-CT in Swiss mice (Figure 4C). Altogether, these results confirm the potential of these constructs to generate a network of cross-reactive functional cytophilic antibodies against all the members of the MSP3 family in diverse mouse genetic backgrounds, similar to that previously observed in sera from humans exposed to malaria [12]. There is a clear improvement in the quality of the humoral response induced against MSP3 antigens by the newly designed PPs, as compared to the first multigenic construct “poly-bcd” previously studied [13], when using the same adjuvant in the same mice strains.

### Antigenicity studies show a dominance of cytophilic IgG in sera of malaria-exposed individuals

**Figure 5:**
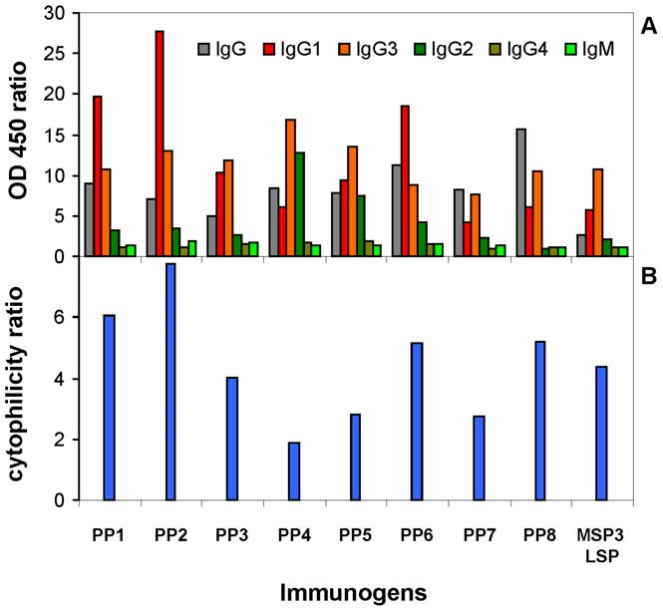
Antigenicity of the PPs with sera from malaria-exposed individuals. (A) Analysis of the antibody isotype responses against the PPs. Results are expressed as mean OD_450_ values. (B) Determination of the cytophilicity ratio (IgG1+IgG3/IgG2+IgG4+IgM) associated to the antibody response to each PP.

We analyzed the reactivity with each PP of naturally acquired IgG present in the sera from a small cohort of malaria-exposed individuals (n = 28, mean age = 18±10.5 years). All the sera from villagers tested recognized each PP, and this recognition was associated with various intensities of the natural IgG responses (Figure 5A). Importantly, these responses were higher in comparison to the MSP3-LSP ninety-six residues long peptide (MSP3.1 aa_154–249_) currently assessed in clinical trials [5], [7], [8], [9]. We observed that all PPs were mainly targeted by human cytophilic IgG1 and IgG3 antibodies with a high CR ranging from 2 for PP4, up to 8 for PP2 (Figure 5B). In addition, antibody response with a higher CR than MSP3-LSP targeted PP1, PP2, PP6 and PP8. Surprisingly the tetravalent construct, PP2, stands out as the most valuable, i.e. more than the hexavalent construct, PP1, in this respect.

We then analyzed the potential relationship between this natural antibody response and the occurrence of malaria attacks recorded over a period of one and three years, during which the mean number of attacks was 1.68±1.91 and 3.46±3.91 respectively. We first performed an univariate analysis showing an association between high cytophilic responses against MSP3-LSP and low number of malaria attacks over a one year period (*R^2^ = 0.208, p = 0.0147*), which remained statistically significant over three years of follow-up (*R^2^ = 0.241, p = 0.0079*). Immune responses against PP1, PP7 and PP8 showed the same association with protection from malaria attacks (*R^2^ = 0.281, 0.223, 0.284*, respectively) as that observed with MSP3-LSP, over a one year period, without increase in significance over three-years of follow-up. Conversely, high CR values did not all correlate with a lower occurrence of malaria attacks, as in contrast no significant association was found for instance with PP2 (*R^2^ = 0.097*). Models of interactions between malaria attacks and available explanatory variables were then tested by multivariate analyses taking into account antibody response levels, age, G6PD deficiency, Hb phenotype and the proportion of time spent in the village during active follow-up. Models of interactions show that the strongest association is found between high anti-MSP3-LSP cytophilic responses and a limited number of malaria attacks over one and three yeas of follow-up (*R^2^ = 0.498, p<0.0001* and *R^2^ = 0.569, p<0.0001*, respectively). An association between high anti-MSP3-LSP cytophilic responses and the occurrence of a limited number of malaria attacks, was found and increased from one to three years of follow-up (with *F = 4.99, power analysis = 0.575; p = 0.03347* and *F = 6.91, power = 0.715; p = 0.01444*, respectively). Among the PP constructs, the model of interactions showed the best fit between high anti-PP8 cytophilic antibody responses and low number of malaria attacks during one year of follow-up (*R^2^ = 0.499 p<0.0001*). Similarly, specific PP8 cytophilic antibody was associated with reduced malaria attacks experience during the first year of follow-up (*F = 5.02; power = 0.577; p = 0.0342*). Other results to other constructs show the same trend though several do not reach significance most likely due to the small size of the cohort studied.

### PP-induced antibodies react with native parasite antigens

**Figure 6:**
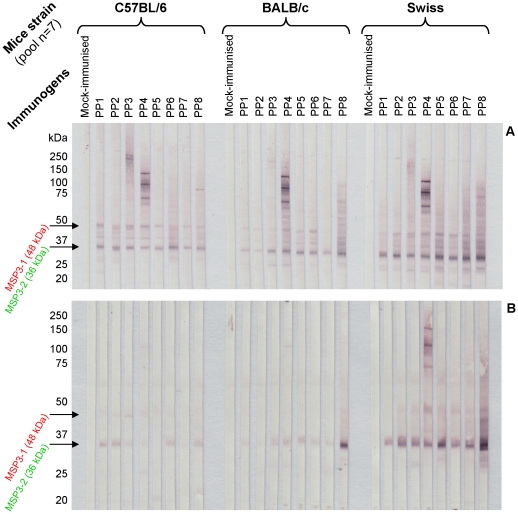
PP-elicited IgG recognize native proteins of the asexual blood stage *P. falciparum*. *Western blotting* of human erythrocytes infected by *Plasmodium falciparum* (3D7 clone) and probed with pooled immune sera from groups of mice two weeks (A) or six months (B) after the sixth immunization with polyproteins or with adjuvant alone (mock immunized).

Immunofluorescence (IFA) and *Western blotting* were used to analyze the ability of PP-induced antibodies to recognize epitopes expressed by native proteins on asexual blood stage parasites. IFA results showed that all PP-induced antibodies in the three mouse strains after the third immunization recognized epitopes on native proteins of *P. falciparum* parasites with titers of up to 1∶8000. Sera from mice immunized with adjuvant alone or LSA1, an antigen exclusively expressed during the parasite liver stages, were unable to recognize parasite asexual blood stages (Figure S4). The specificity of the antibody response was further characterized by western blotting where IgG from immune sera sampled two weeks after the third immunization with PPs showed a strong reactivity with MSP3 proteins (Figure 6A). Such specific IgG binding was mostly directed against two proteins corresponding to MSP3.1 at 48 kDa and MSP3.2 at 36 kDa, and this was confirmed by competition experiments carried out with the corresponding soluble recombinant MSP3 peptides, respectively (data not shown). The specificity to the other discrete MSP3 bands could not be assigned to a particular MSP3 antigen. PP3 and PP4 induced antibodies specific for the p27 and LSA3 peptides, respectively. No antigen recognition was detectable with sera from mice immunized with adjuvant alone or LSA1.

The PPs induced a long-lasting anti-MSP3 response, detectable six months after the last injection, and antibody reactivity was almost entirely directed against MSP3.2, pointing to this MSP3 family member as one with unique features (Figure 6B). Immune sera from outbred Swiss mice were the most reactive against the native MSP3 antigens, particularly during the memory phase of the response. PP8 stands out by eliciting a markedly stronger antibody response against the native MSP3.2 antigen, as seen in sera from BALB/c and Swiss strains collected six months after the last immunization. The reasons for this observation are not clear, as one would expect that larger PPs would induce similar responses.

### PP-elicited antibodies inhibit *in vitro* growth of *P. falciparum* in ADCI assay

**Figure 7:**
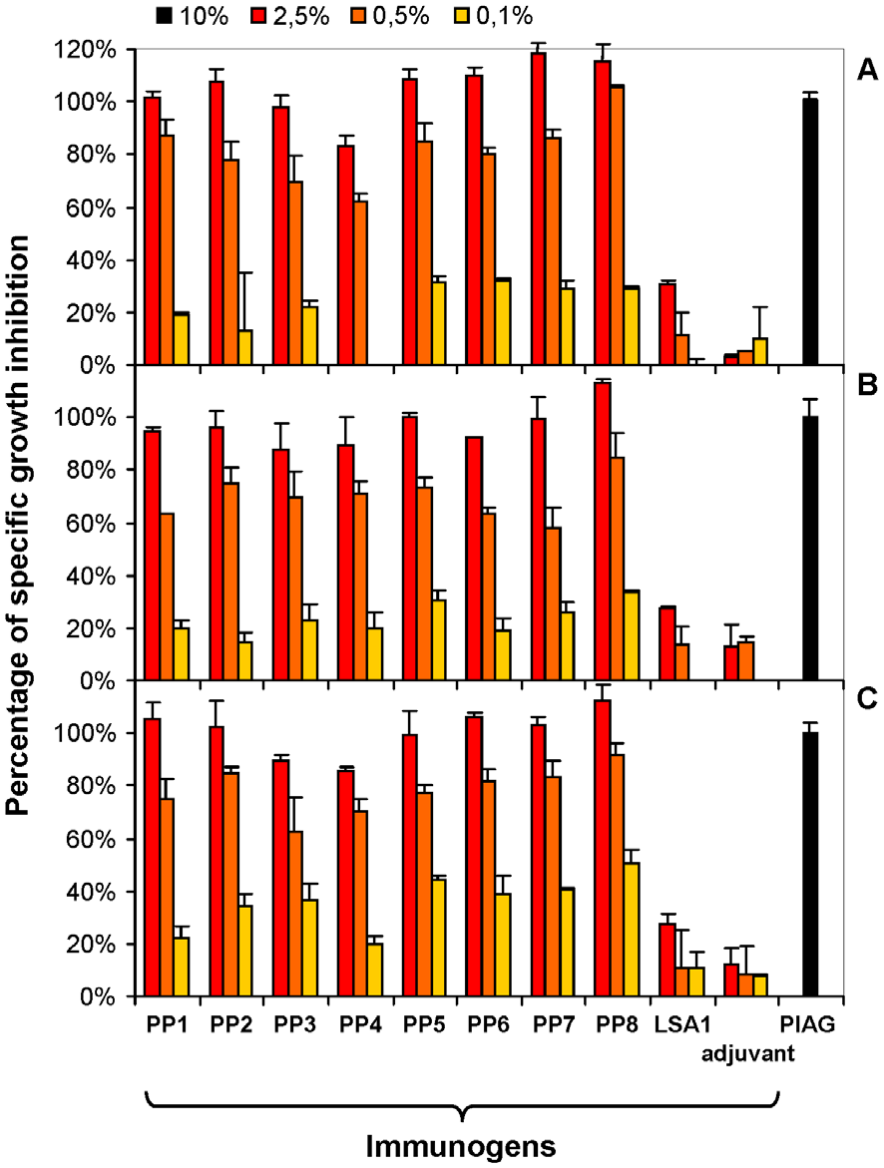
PP-induced antibodies inhibit *in vitro* the growth of the asexual blood stage of *P. falciparum* in ADCI. ADCI was performed with human MN and parasites cultured for 4 days with immune sera from C57BL/6 (A), BALB/c (B) and Swiss (C) mice harvested two weeks after the fifth immunization with the MSP3-PPs. Sera from mice immunized with an irrelevant *P. falciparum* antigen (LSA1) or with the adjuvant alone, were used as negative controls. A pool of immune sera from individuals living in endemic areas (PIAG) was used as positive control. The PIAG was used at a dilution of 10%, the mice immune sera were used at a 5-fold serial dilution of 2.5%, 0.5% and 0.1%. Results are expressed as adjusted SGI values compared to the PIAG value that was redressed to 100% of SGI effect.

*In vitro* inhibition of asexual blood stage parasites by PP-induced antibodies was assessed in ADCI assay using sera collected after the third immunization for some mice strains (Figure S5) and after the fifth injection for all strains (Figure 7). As negative control, we used LSA1, a liver stage antigen eliciting also a majority of cytophilic antibodies but having no significant ADCI activity. In addition, no significant direct inhibitory effect (<15% parasitic growth inhibition) of parasite growth by antibodies alone was observed. The observed anti-parasitic activity in monocyte-dependant fashion was strong and homogeneous using sera from all PP-immunized groups from the three strains of mice already after the third and later after the fifth immunization. The inhibitory effect of induced antibodies was as high as, or higher than, that obtained using the pool of African immunoglobulins (PIAG) even though mice sera were assessed at a four-fold lower concentration (2.5%) than the PIAG (10%). This PP-specific anti-parasite activity remained substantial when sera were used at 0.5% concentration (diluted 200 fold) but decreased notably at 0.1%. No significant ADCI effect was observed with sera from mice immunized by LSA1 or by the adjuvant alone. As a further example of the potency of the antibodies elicited by the constructs, the ADCI activity was similar using the PP-immunized mice sera at a 10 fold lower concentration than affinity purified human anti-MSP3.1b IgG [6].

## Discussion

We report here the design and analysis of the immunological properties of eight PPs combining one or several peptides belonging to the MSP3 family of proteins. The reactivity pattern of these new vaccine constructs showed that: **a)** they induced, in genetically distinct mice, antibodies that recognize *P. falciparum* antigens and native parasite proteins from asexual blood stage with a marked prevalence of cytophilic IgG subclasses; **b)** they favoured the induction of cross-reactive antibodies targeting the structurally related MSP3 epitopes; **c)** they all bound natural IgG from sera of malaria-exposed people with a predominance of specific cytophilic IgG subclasses; **d)** PP-induced antibodies exerted a strong inhibitory effect on the *in vitro* growth of *P. falciparum*, more potent than that obtained with the first MSP3-LSP construct designed.

Our interest for this multigene family and for vaccine constructs deriving from its different members originates from the protection elicited in African children by MSP3.1 [9], and from the very unusual features of this multigene family [11]. The six C-terminal regions are all expressed by each parasite, have a very similar organisation, are all remarkably conserved in different *falciparum* strains and they are targeted by broadly cross reactive cytophilic antibodies effective in the MN-dependant ADCI mechanism. Given these features, it was considered logical to produce vaccine constructs by assembling the most representative regions from the C-term of those genes under the main hypothesis that vaccines generating the same wide range of diverse antibody species should contribute to improved biological anti-parasite activity.

Vaccine constructs targeting different antigens at different stages of the parasite cycle are considered as attractive combinations in view of both the great genetic variability and complexity of the parasite life cycle, illustrated by the extensive repertoire of various proteins expressed by blood schizonts [31]. The development of an effective multi-epitopic malaria vaccine is currently under investigation by different research groups with promising results displayed in animal models. Several of these studies usually associate either two distinct antigens from two different stages of the life cycle, or two different antigens from the same stage. This is a markedly different approach as compared to ours, as each of the epitopes selected in our multigene constructs was chosen based on detailed analyses [1], [6], [10], [12], [13] exclusively relying on indications originating from natural interactions between human hosts and *falciparum* parasites, and also because the constructs contained antigens from structurally related genes [2], [11].

In our constructs, the aim was to diversify the response of B-cells and T-helper cells to a repertoire of epitopes from a single gene family showing epitope redundancy. Hence, the goal was to improve immune responses towards similar targets and not to combine immune responses to different target antigens. The resulting value of the constructs was analyzed at the pre-clinical level in experimental rodent models. Whereas a first vaccine candidate has already gone far in its clinical development, the goal of our approach was to develop a second generation of MSP3-derived vaccine with improved features.

The present eight PPs designed to increase the number of ADCI targets were easily expressed in *Escherichia coli*, and purified as soluble recombinant proteins retaining their antigenicity. Both immunogenicity and antigenicity data indicate that the critical epitopes contained in PPs have not been altered by the construction, are accessible to antibodies, and stimulate the desired subclasses of antibodies.

Immunogenicity was the first criterion to satisfy. Immunizations of three genetically distinct mouse strains with the PPs induced high and long lasting humoral response against MSP3 antigens. This specific response was induced rapidly, usually after a single immunization with peak intensity generally observed after the third immunization. Immunogenicity data in three rodent species used herein, showed no evidence for major genetic restriction, i.e. overall homogeneous responses with relatively minor differences.

The second important criterion, induction of antibodies with a dominance of cytophilic IgG subclasses, was also fully satisfied. Though there were understandably a range of CR values from one construct to the other, in diverse breeds of mice, and in malaria exposed humans, the important point is that in all cases there was a dominance of cytophilic humoral responses, as shown by a CR>1. Our results demonstrate that a majority of murine cytophilic IgG subclasses (namely, IgG2a, IgG2b and IgG2c) were elicited after three immunizations as expressed by the cytophilicity ratio values attesting of a favourable isotypic balance. Indeed for a mechanism such as ADCI which implies a triggering of monocytes trough their Fc-γ receptors, the “quality” of the antibody response directed against the members of the MSP3 family is an important criterion. This may indirectly indicate that the constructs included T-helper cell epitopes effective in diverse genetic backgrounds. The prevalence of cytophilic IgG subclasses, i.e. able to bind to Fc-γ receptors expressed at the surface of monocytes is in keeping with the induction of a Th-1 response as was already reported for the first multigenic construct which induced high levels of IFN-γ [13]. Other reports have shown that immune responses skewed toward a Th1-like response are involved in the production of murine IgG subclasses, notably IgG2a [32], [33], [34]. Incidentally, our results also stress the importance of investigating the IgG2c isotype, particularly in C57BL/6 mice, by using secondary antibodies that are specific for this rather unusual IgG subclass.

A third important goal reached was the generation of a broader immune response directed at several, or nearly all, members of the MSP3 protein family. Indeed, by combining a number of epitopes belonging to the same family of proteins, we could extend the reactivity of elicited antibodies to other related epitopes, not contained in the immunogens. This observation proved true for the majority of the six MSP3 antigens in two out of the three genetically distinct mice tested. However, in the majority of cases, by combining selected epitopes originating from the antigenic motifs of a conserved protein family, it proved possible to induce high levels of cross-reactive antibodies targeting several well-conserved epitopes including both those with sequence identity and those with sequence divergences.

The last goal reached was to induce antibodies with a satisfactory functional activity *in vitro* against asexual blood stage parasites of *P. falciparum*. ADCI assays demonstrated the ability of PP–elicited antibodies to react with native parasite antigens and to cooperate with MN to achieve parasite killing. Importantly this antiparasitic activity was observed for antibodies induced by all of the constructs. No enhancement was observed. Furthermore, anti-PPs antibodies exhibited a strong effect, similar or higher than that obtained using the PIAG, *i.e.* the pool of sera which following passive transfer reduced parasitaemia in malaria patients [35]. The growth inhibition effect remained significant even at low concentration i.e. when sera were diluted 200 fold. It is worth noting here that the capacity to inhibit *in vitro* the growth of *P. falciparum* asexual blood stages using the ADCI assay was stronger than that found with the antibodies produced by the vaccine candidate MSP3-LSP. The specific parasite growth inhibition associated with sera from mice immunized with the PP8 was the highest, remaining quite significant even with sera diluted 1,000 fold (i.e. diluted one hundred-time more than the PIAG). This last observation is in agreement with a previous study reporting the value of MSP3.2 as vaccine candidate [36].

Antigenicity studies with sera from endemic area populations bring support to the relevance of mouse data to the human situation. Indeed, naturally occurring IgG recognized the various constructs with predominance of cytophilic antibodies. Furthermore, in these sera, reactivities observed against the PPs displayed similar or higher prevalence and IgG response than against the individual epitopes [12].

Over-immunization by giving three additional injections after the first three, i.e. six in total, was used as a mean to investigate the possibility that immunoregulatory regions were included in the constructs. Indeed, using the whole C-term region of MSP3.1 we observed previously that over immunization induced a sharp decrease in antibody titers as compared to those obtained after three immunizations, particularly in C57BL/6 mice [13]. This effect was found related to the inclusion of the “e–f” peptide region in the immunogen. Although, no attempt was made to characterize the nature of this regulatory mechanism nor to identify the epitopes involved in this effect within those regions, this observation nevertheless led us to choose the MSP3-LSP in which the extreme C–term region (covering peptides “e” and “f”) prone to induce a down regulation of immune responses is excluded. Results from mice receiving six consecutive immunizations of our constructs, did not show any decrease in antibody responses, with any of the constructs studied, indicating they are not prone to induce the regulatory effect previously observed with MSP3-1 C-term region. Results show that using a similar immunization protocol, i.e. with the same adjuvant and in the same mouse strains, the new vaccine constructs induced antibody responses similar in three different mouse genetic backgrounds, and higher titers as compared to what was found using the first generation, MSP3-LSP as well as the first MSP3-derived multigene construct [13].

Hence, most constructs were effective, and nearly all satisfied the criteria of immunogenicity, antigenicity, cytophilicity, and antibody functional activity. However, this was, to a certain extent, unexpected. In spite of their satisfactory features, the newly designed PPs do not enable us to single out a particularly “better” vaccine construct from the group of eight, with superior features compared to the others, to be taken forward into clinical development.

Avidity studies did not distinguish either the antibodies elicited by each PP. The avidity of antibodies elicited by the PPs was indirectly evaluated in sera sampled after the third and the fifth immunization, by ELISA using an asexual blood stage extract of *P.falciparum*, in the presence of increasing concentrations of the chaotropic ion NH_4_SCN. Results (not shown) indicate that 50% of the reactivity of PP specific IgG to native antigens was removed by 1 M and 1.5 M NH_4_SCN in sera taken after the third immunization, and after the fifth immunization, respectively. These results indicate relatively high avidities of PP-induced antibodies, which increase with the number of immunization stressing thereby the occurring immunologic selection in favour of antibodies with better-fitted paratopes. Results were similar in each of the three mice strains (not shown). Thus, the IgGs induced by each of the eight PPs displayed similar patterns of avidity in the assay employed.

The addition of coiled-coil fragments from different proteins had no significant effect. These were added with two goals. The first one being to improve overall structure of MSP3 antigens, with the implication that it may improve immunogenicity, the other being to combine responses against MSP3 and against repeat domains from other vaccine candidates (namely, antigens from asexual liver and blood stages, LSA3 and p27 respectively). Available results do not indicate that either of those hypotheses was true. Responses to MSP3 antigens was as good as but not better than without the additional antigens.

It is well known in the malaria vaccine development field that mouse immune responses frequently differ from those of human volunteers leading in phase I trials to an immunogenicity of vaccine prototypes frequently different to that obtained in preclinical models. Therefore, even if most vaccine constructs satisfied the majority of our criteria in preclinical conditions, it would be required to address the immunogenicity and particularly the duration of functional immune response in a phase I trial where two or three selected constructs would be investigated head to head. Given the above results, our choice of candidate molecules that should be taken forward to clinical trials, would include two, if not three of the PPs selected based on the assays reported herein. Based on the preclinical data and the clinical results obtained with MSP3-LSP, it is anticipated that each of those three should be immunogenic in humans as was seen across three mouse strains. Criteria for such a phase Ia study would be the titers, cytophilicity ratio and above all, duration of functional immune responses in humans.

## Supporting Information

Figure S1 **Quality control of the production in** ***E. coli*** **of the recombinant PPs.** Electrophoresis of one microgram of the purified PPs on a 10% acrylamid gel under denaturing conditions and Coomassie blue staining (A). *Western blotting* of the PPs probed with a rat anti-MSP3.1 immune serum at a 1∶100 dilution (B).
(TIF)

Figure S2 **Induction by PPs of specific IgG2a against MSP3-CT antigens.** Determination of specific murine cytophilic IgG2c against the MSP3-CT antigens, two weeks after the third injection of 20 µg of immunogens adjuvanted in Montanide ISA720. Results were expressed as the geometric mean of the OD_450_ obtained for the single dilution of sera at 1∶1000.
(TIF)

Figure S3 **Induction by PPs of specific IgG2b against MSP3-CT antigens.** Determination of specific murine cytophilic IgG2a against the MSP3-CT antigens, two weeks after the third injection of 20 µg of immunogens adjuvanted in Montanide ISA 720. Results were expressed as the geometric mean of the OD_450_ obtained for the single dilution of sera at 1∶1000.
(TIF)

Figure S4 **Binding of PP-induced IgG to native proteins from asexual blood stages parasites of** ***P. falciparum*** **.** Immunofluorescence analysis with immune sera from BALB/c mice immunized with adjuvant alone (upper left); PP1 (upper right); PP8 (lower left) and LSA1 (lower right) collected after the third immunization. Staining on acetone-fixed thin smear of very mature stages asexual blood stages from *P. falciparum* (3D7 clone) was made with murine immune serum used all at 1∶1000 final dilution.
(TIF)

Figure S5 **Anti parasitic activity of PP-induced antibodies in ADCI against** ***P. falciparum*** **asexual blood stage parasites.** *In vitro* growth inhibition of *P. falciparum* parasites (3D7 clone) cultured in presence of human monocytes and immune sera from C57BL/6, BALB/c and Swiss mice harvested two weeks after the third immunization with PPs. Sera from BALB/c mice immunized with non relevant *P. falciparum* antigen (LSA1) and with adjuvant alone, were used as negative controls. A pool of immune sera from individuals living in endemic areas (PIAG) was used as positive control. The PIAG was used at a dilution of 10%, the mice immune sera were used at a serial dilution of 2.5%. Results are expressed as adjusted SGI values compared to the PIAG value that was redressed to 100% of SGI effect.
(TIF)
