# Supplementary figures and images for: Pre-Clinical Assessment of Novel Multivalent MSP3 Malaria Vaccine Constructs
Source: PLoS One. 2011 Dec 1;6(12):e28165. doi: 10.1371/journal.pone.0028165 (PMC3228738; doi:10.1371/journal.pone.0028165)

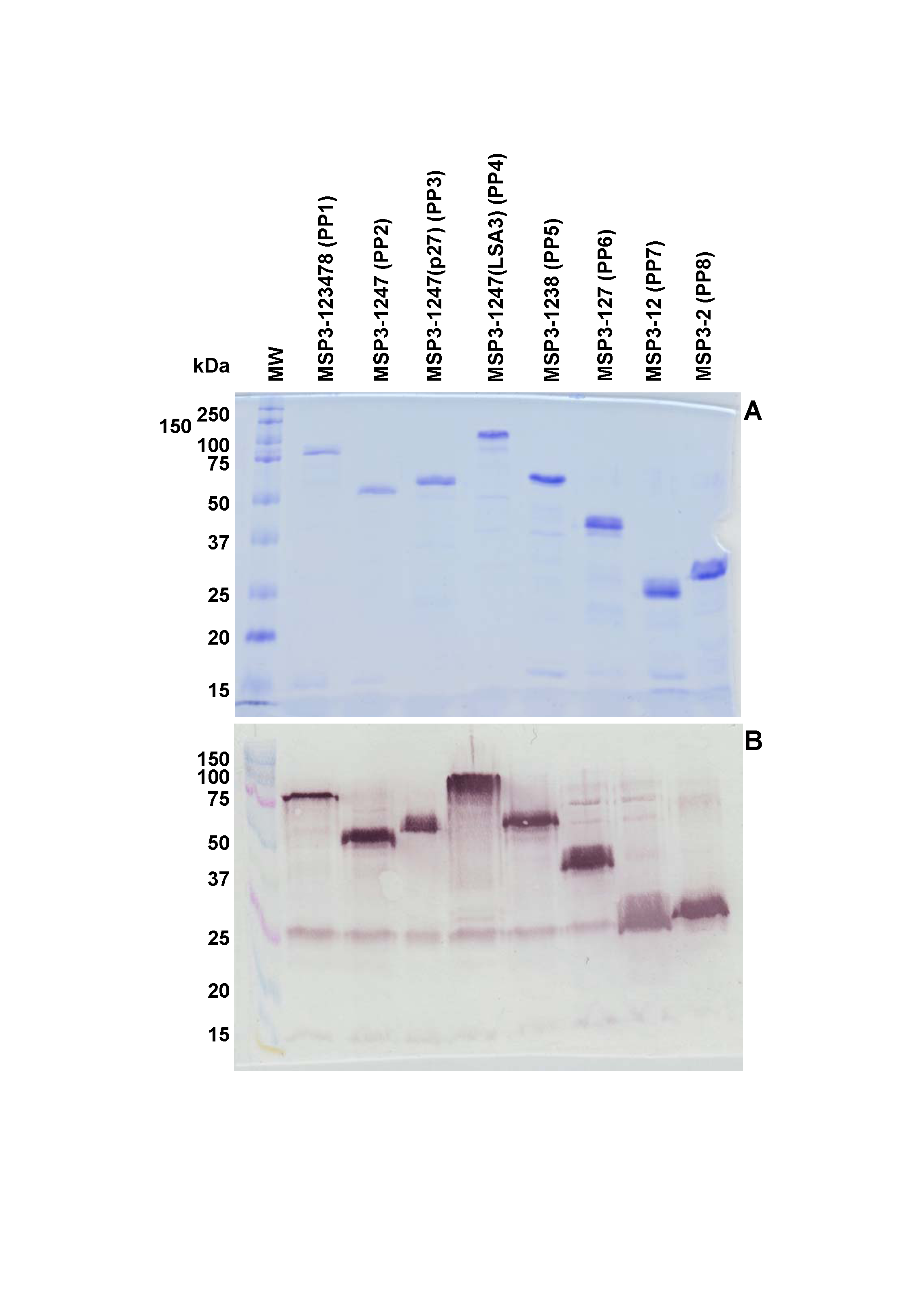

Supplement: Figure S1 — Quality control of the production in E. coli of the recombinant PPs. Electrophoresis of one microgram of the purified PPs on a 10% acrylamid gel under denaturing conditions and Coomassie blue staining (A). Western blotting of the PPs probed with a rat anti-MSP3.1 immune serum at a 1∶100 dilution (B). (TIF) [file pone.0028165.s001.tif]

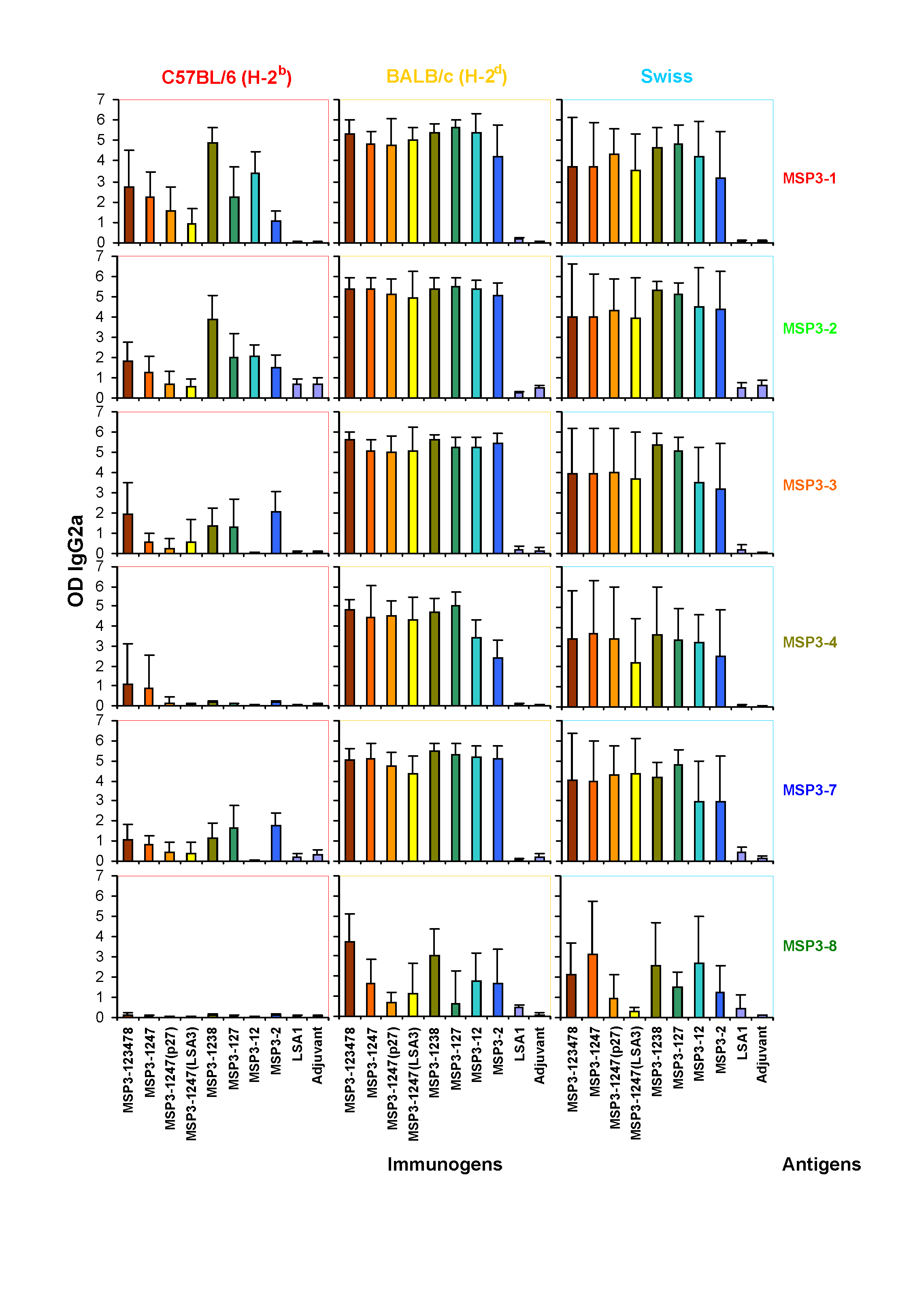

Supplement: Figure S2 — Induction by PPs of specific IgG2a against MSP3-CT antigens. Determination of specific murine cytophilic IgG2c against the MSP3-CT antigens, two weeks after the third injection of 20 µg of immunogens adjuvanted in Montanide ISA720. Results were expressed as the geometric mean of the OD450 obtained for the single dilution of sera at 1∶1000. (TIF) [file pone.0028165.s002.tif]

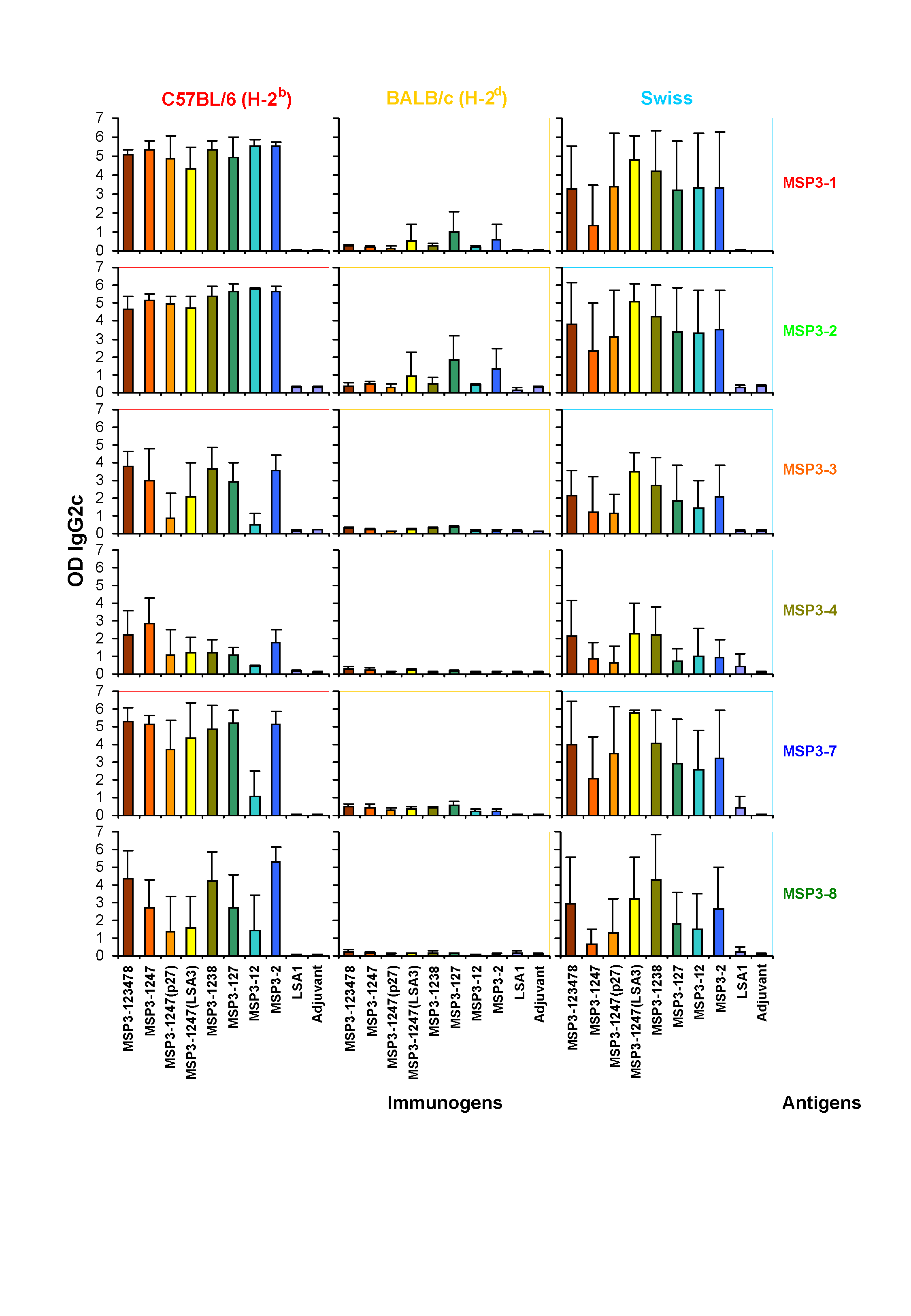

Supplement: Figure S3 — Induction by PPs of specific IgG2b against MSP3-CT antigens. Determination of specific murine cytophilic IgG2a against the MSP3-CT antigens, two weeks after the third injection of 20 µg of immunogens adjuvanted in Montanide ISA 720. Results were expressed as the geometric mean of the OD450 obtained for the single dilution of sera at 1∶1000. (TIF) [file pone.0028165.s003.tif]

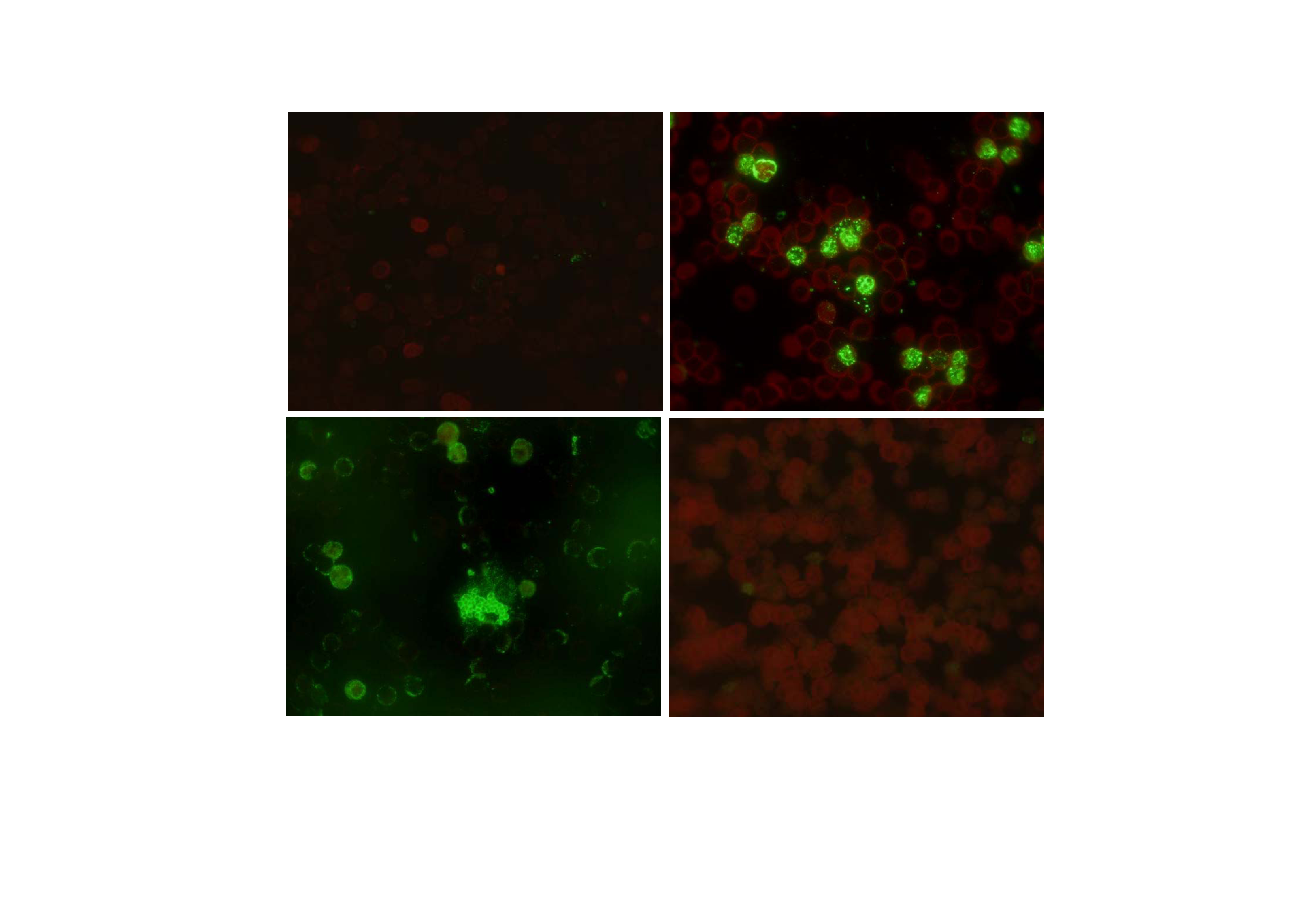

Supplement: Figure S4 — Binding of PP-induced IgG to native proteins from asexual blood stages parasites of P. falciparum . Immunofluorescence analysis with immune sera from BALB/c mice immunized with adjuvant alone (upper left); PP1 (upper right); PP8 (lower left) and LSA1 (lower right) collected after the third immunization. Staining on acetone-fixed thin smear of very mature stages asexual blood stages from P. falciparum (3D7 clone) was made with murine immune serum used all at 1∶1000 final dilution. (TIF) [file pone.0028165.s004.tif]

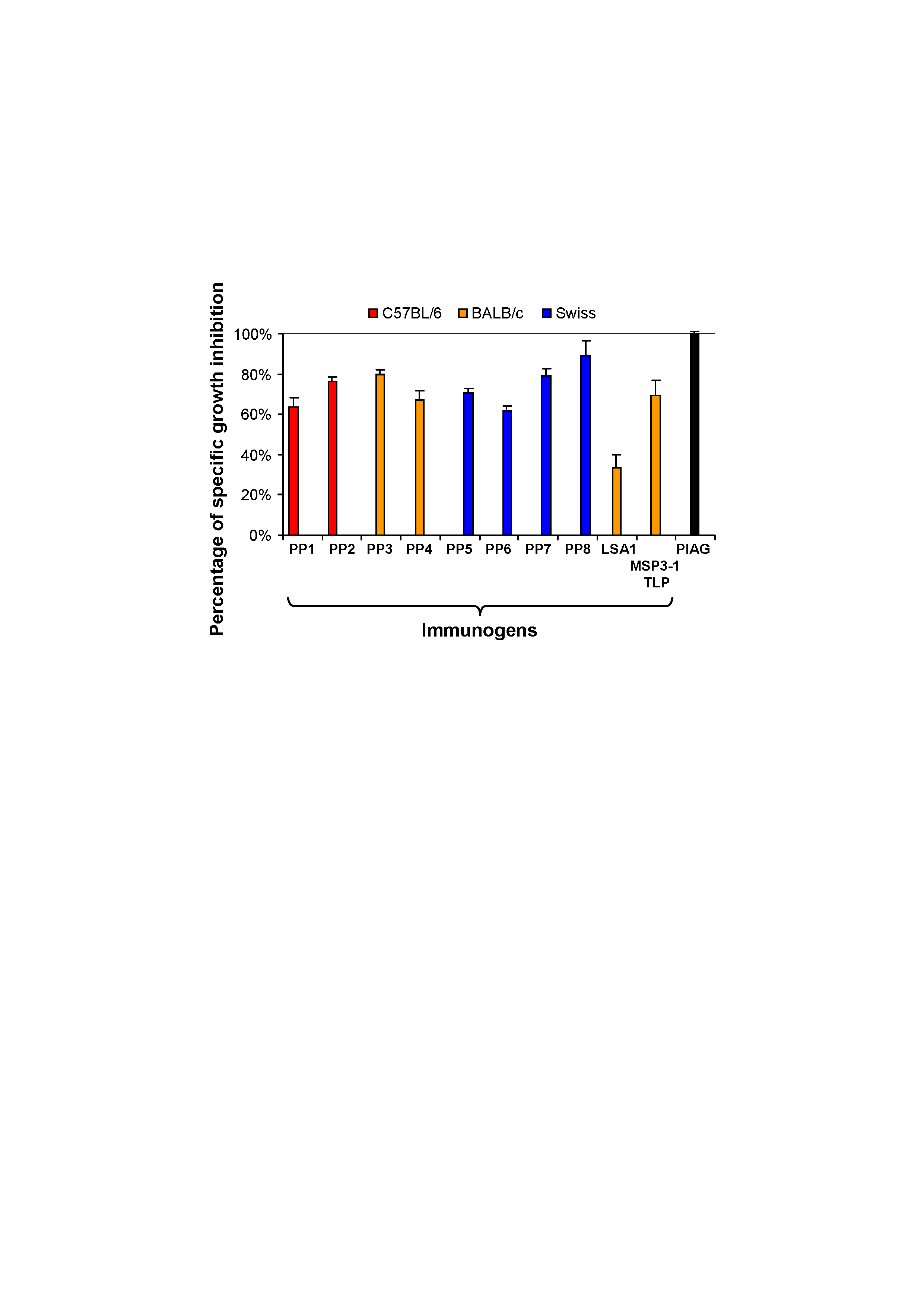

Supplement: Figure S5 — Anti parasitic activity of PP-induced antibodies in ADCI against P. falciparum asexual blood stage parasites. In vitro growth inhibition of P. falciparum parasites (3D7 clone) cultured in presence of human monocytes and immune sera from C57BL/6, BALB/c and Swiss mice harvested two weeks after the third immunization with PPs. Sera from BALB/c mice immunized with non relevant P. falciparum antigen (LSA1) and with adjuvant alone, were used as negative controls. A pool of immune sera from individuals living in endemic areas (PIAG) was used as positive control. The PIAG was used at a dilution of 10%, the mice immune sera were used at a serial dilution of 2.5%. Results are expressed as adjusted SGI values compared to the PIAG value that was redressed to 100% of SGI effect. (TIF) [file pone.0028165.s005.tif]
